# General Report on the Sanatory Condition of the Labouring Population of Great Britain

**Published:** 1843-04

**Authors:** 


### Art. IV.

1. *General Report on the Sanatory Condition of the Labouring Population of Great Britain.* By Edwin Chadwick, Esq.—*London,* 1842. 8vo, pp. 457. *Printed for Her Majesty's Stationery Office.*
2. *Local Reports on the Sanatory Condition of the Labouring Population of Great Britain, in consequence of an Inquiry directed to be made by the Poor Law Commissioners.* Two Vols. 8vo.—*London,* 1842. Vol. I.—*England and Wales,* pp. 444. Vol. II.—*Scotland,* pp. 334. *Printed for Her Majesty's Stationery Office.*

The readers of this Journal have, more than once, been called on by us to bestow their approbation and applause on the scientific labours of a gallant gentleman not of the medical profession—we refer to Major Tulloch—whose Reports on the Health of the Army would do honour to any physician, and must, indeed, be ever ranked among the most valuable documents in the department of statistical medicine. In the works before us we have the results of the labours of a lay civilian in the same general line of inquiry; and we can bestow no higher praise upon them—nor, indeed, characterize them more justly—than by saying that the Sanatory Reports are worthy of being classed in the same high category as Major Tulloch's Army Reports. In the conception and origination of the inquiry, of which these reports are the fruits, Mr. Chadwick gave unequivocal proof of his being influenced by genuine philanthropy; and in conducting it to its issue in the works now before us, he has shown a corresponding degree of industry and acuteness, and comprehensiveness of mental grasp.

It appears that in May 1838 the Poor Law Commissioners presented to Lord John Russell a report "relative to certain charges which have been disallowed by the auditors of unions in England and Wales;" together with two supplementary reports. One of these was by Dr. Arnott and Dr. Kay, on the prevalence of certain physical causes of fever in the metropolis, which might be removed by proper sanatory measures; the other, by Dr. Southwood Smith, was on the condition of the poor in Bethnal Green and Whitechapel, and exemplified some of the physical causes of sickness and mortality to which the poor are peculiarly exposed. These reports were appended to the Fourth Annual Report of the Commissioners, being Appendix A, No. 1.

On the 29th April, 1839, the Commissioners received a Report from Dr. Smith on the prevalence of fever in twenty metropolitan unions or parishes during the year ending 20th March, 1838. This constituted No. 2 of Appendix C in the Fifth Annual Report of the Commissioners.

In August 1839 the Poor Law Commissioners received instructions from the Secretary of State for the Home Department to inquire to what extent the causes of diseases mentioned in the reports just named prevailed amongst the poor or labouring classes in England and Wales. They were thus constituted a board of health; not, however, for administration but for inquiry; and, consequently, in November following, the whole administrative machinery of the Commissioners throughout England and Wales was put in motion for the purposes specified. In January 1840 instructions were issued from the Home Office to the effect that the inquiry should be extended to Scotland. These extensive inquiries terminated in the presentation to Parliament, at the end of the last session, of the Reports we now propose to consider.

The General Report is a condensation of the local reports, together with a general report from the Commissioners, and we do not hesitate to say that it constitutes the most valuable and complete treatise on certain departments of medical police ever published, either in this or any other country.

The principles of medical police have been acted upon in England from a very early period; certainly 600 years ago. But, in fact, these principles may be traced in all societies of men, however primitive; the adoption of them may be termed an instinct. The common law against nuisances contains the kernel of our medical police, the term nuisance meaning anything by which the health or the personal safety, or the conveniences of the subject might be endangered. Thus it is contrary to law to divide a messuage in a town for poor people to inhabit, by which it will be more dangerous in time of infection. Mr. Chadwick says that the common law obligation upon all owners of property has, in general, been adhered to by the superior courts, "*Prohibetur ne quis faciet in suo quod nocere possit in alieno; et sic utere tuo ut alienum non lædas.*" The plain English of this is "do as you would be done by," a precept continually set at nought by the operation of the antagonist principle of selfishness—"every man for himself and God for all." Legislative enactments in great variety have been adopted to counteract this ever-operating desire to seek individual benefit at the cost of the public health and comfort. Such, however, has been the ignorance of all classes (not excepting the medical profession) of the leading principles of state medicine that these enactments have always been crude and insufficient, and generally useless. As medical science became more cultivated, hygiènic knowledge was more diffused, and we find an infinity of local acts and municipal bye-laws providing for the supply of water and the sewerage of towns; the cleansing of streets; the discovery and destruction of unwholesome food and drink, &c. Boards of health were established in Ireland, but have utterly failed in achieving any permanent good. The most important authority for removing nuisances was vested in the courts leet. Juries are empanelled at these courts and authorized to perambulate districts and judge of nuisances upon the view. They have been discontinued in many parts of the country since the new Poor Law came into operation, and it would be as well if they were discontinued altogether, as in places where they are still held, the "annoyance juries" are composed of petty tradesmen and mechanics, utterly ignorant, we need not say, of the aim and scope of their duties, and of the knowledge necessary to their proper discharge. The mode in which a metropolitan "annoyance jury" proceeds is thus described by two respectable witnesses who were unexpectedly called upon to serve. We quote from Mr. Chadwick's Report:

"When we were sworn in we went over the district; we went through many places which were disgustingly filthy, that I have since learned were places where there is always fever, but we are not told about it; the afflicted knew nothing of our coming, and we had no medical officer or means to enable us to detect the presence of any nuisances which would endanger the public health. The number of persons sworn in was twenty-four, of whom I can remember six were publicans, (at one or other of whose houses we dined on the days of meeting,) one or two cheesemongers, three or four tailors or drapers, one builder, and one bricklayer. .......... None of the jury knew the nature of their duties further than that they were to examine weights and measures; that part of their duty respecting the removal of nuisances, or of things affecting the health or lives of the inhabitants of the district which we perambulated, was entirely neglected or lost sight of."..........

The witness goes on to say, however, that condemnation was pronounced on an old house, on the fiat of the builder and bricklayer. The utter inefficiency of these courts leet was clearly exhibited when the cholera was epidemic. The boards of health, we may observe, which were then instituted to replace these courts were little less useless.

There is a self-regulating principle in the social and moral world, as well as in the physical. Ideas, if truthful, always end (sooner or later) in action. As science became diffused more minds were rendered capable of appreciating the warnings given from time to time by philanthropic physicians. The seeds sown by these patient and unrewarded, nay sometimes persecuted, well-doers, were not sown in vain. And although from a want of system it would be difficult to trace their effects to the proper sources, these effects are exhibited in a diffusion through this country of the principles and practice of public hygiène more extensive and more efficient than is to be found in any other country. For several years scarcely a single session of the Imperial Parliament has terminated without something being done for the public health. Quarantine, vaccination, the supply of water to towns, sewerage and drainage, boards of health, the relief of lunatics and of the sick poor; the state of burial grounds; the health of emigrant ships and of prisons; the inspection of factories; drunkenness; medical education, and numerous other minor heads of the general subject, might be mentioned in proof of this statement. All the proceedings of this kind instituted by Parliament have been unconnected and clumsy. In the system of moving for returns and select committees there was simply nothing more than a cumbrous improvement in the courts leet, and the members of the committee were scarcely more acquainted with the principles of hygiène than the "annoyance" jurymen. In one thing, however, they were superior, namely, in having the power in some degree to acquire knowledge from competent individuals. Yet when these individuals were heard as witnesses, the members of the committee were too often in the ridiculous position of persons utterly ignorant of a science sitting in judgment on the opinions of the learned and experienced; appearing more ludicrous, indeed, to the initiated, than the whole tribe of justice Shallows.

This incapacity to conduct hygiènic inquiries with effect was accompanied by an utter want of unity of purpose and system. Reports were never acted on; or worse, were considered mere collections of garbled evidence got together for a particular purpose, and not therefore to be relied on. The labours of one session were forgot by the next. A return made on the motion of a member was no sooner made than forgotten, or laid by as waste paper. An instance of this is before us. On the motion of Mr. Hume a return was made of the places of burial within the bills of mortality, belonging to each parish or precinct under the authority of the Bishop of London; their locality, extent by actual admeasurement, and the number of burials in them in each of the years 1830, 1831, 1832, and in 1833 to 1st June. These particulars respecting 107 metropolitan burial grounds were obtained, with plans of several; yet only nine years after, a select committee sits to inquire into the same subject, and reports, (we have reviewed the Report of this committee in our present Number,) without, apparently, being aware that such a document existed.

The government and legislature have, however, practically acknowledged that their previous plan was defective by the appointment of various commissions of inquiry, and subsequently of administration. Or it may be that the great accumulation of public business, the vast extent of the empire, and the multifarious duties incumbent on the legislature, especially on the House of Commons, compelled the adoption of such a course. However this may be, the poor-laws, public charities, and employment of children in factories and mines; medical charities, the health of towns, and other matters connected with public hygiène, were investigated by these commissions with much improved results. Subsequently an improvement was even made on these commissions, for it being found that the objects of inquiry allotted to each commission were often cognate, the commissions were virtually consolidated by employing the same individuals on several commissions. This consolidation of duties is most systematic and obvious in Ireland, where the commission system has always been most rife; but the Reports before us are a proof that it is gaining ground in England. The framers of the Act of Parliament which constituted the Poor Law Commission never contemplated that that commission should become a board of health.

The advantages of this system over the old one are obvious. The commissioners by devoting their whole time to their subject acquired both knowledge and experience. Besides some were qualified by previous education and habits for the performance of their duties; as, for example, the physicians who constituted a part of the Factories' Inquiry Commission, or those who were appointed to inquire into the medical charities of Ireland. Yet examples are not wanting to prove the necessity of having individuals to perform these public duties thoroughly trained by education and practice. We will mention one of these, as both illustrating our meaning, and illustrating also other points connected with state medicine. In the second report of the Factories Inquiry Commission is a report from Mr. Tufnell. We know nothing more of that gentleman than what we have learnt from his contributions to the blue books of his commission; it is, therefore, from his *res scriptæ* we infer that he is a warm advocate of large factories. As a proof of their superiority he observes:

"Mr. Ashton can afford to pay for all the surgical assistance that is required by his 1173 workmen, as he can contract for it at six guineas a year. Did he employ only a twelfth part of that number he assuredly could not get a surgeon to take the contract at a twelfth part of six guineas. Mr. Bott undertakes to attend to all the ailments of the operatives in Messrs. Lichfield's mills on payment of a half-penny each weekly; he certainly would refuse to attend twenty persons for tenpence a week." (D. 2, p. 207.)

He certainly would do no such thing. Five minutes' calculation would have convinced Mr. Tufnell that these two cases were utterly dissimilar; and having Dr. Mitchell's tables (to which we shall presently allude) before him when he penned that report, he ought to have calculated the real value of the "surgical attendance" contracted for by Mr. Ashton. If Mr. Ashton thought proper to deduct the sum of one halfpenny per week from the wages of his workpeople for surgical attendance he would get in one year the sum of £127 1*s.* 6*d.*, and make a clear profit out of his unfortunate "surgical" drudge of £120 15*s.* 6*d.*, that being the precise difference between the payment of Messrs. Lichfield's operatives to Mr. Bott and of Mr. Ashton to his surgeon. Stating the population of Manchester in round numbers at 400,000, the rate of payment for medical attendance adopted by Messrs. Lichfield's operatives would amount, on the whole population, to £43,330 per annum. So much for the analogy of the two cases. Let us now examine the work and labour done for six guineas per annum. In the supplementary report of the Factories' Inquiry Commission, Dr. Mitchell, the actuary, gives tables from which we can learn the number of attacks of sickness, and the duration of each attack, occurring to persons employed in cotton factories. We quote Mr. Macculloch's condensation of these tables:

| *Working in Cotton Factories, Lancashire.* | *Annual Attacks of Sickness, per cent.* | *Mean Duration of Attacks in Days.* |
|---|---|---|
| Males, ages 11 to 31 | 27·8 | 16·43 |
| Females, ages 11 to 31 | 41·7 | 12·63 |
| Average of both sexes | 41·7 | 14·53 |

So that Mr. Ashton's 1173 workpeople would experience 407·61 attacks of sickness during the year, the average duration of each attack being 14·53 days; and the unfortunate surgeon got nearly threepence halfpenny for each case; or at the rate of six guineas for attending one person constantly sick for rather more than sixteen years. These calculations may appear incredible, but they are really favorable to Mr. Ashton. If his people had no better health than the *picked* labourers in the East India Company's service, the period last mentioned should have been stated at twenty-two years and a half; or if their health be compared with that of the government labourers in the Chatham Dockyard, it should have been estimated at more than twenty-seven years. Six guineas for twenty-seven years' attendance. Two ounces of Epsom salts per week, at one halfpenny per ounce, would for that period cost £5 17*s*. Perhaps we ought not to blame Mr. Tufnell for this slip in his report; he has acted only like other people. The desire for medical knowledge and skill exhibited by government and public bodies has always been accompanied by a desire to get it for nothing—or less; as in the case just analysed. What we wish to insist upon is, that if Mr. Tufnell had been regularly educated and trained for the performance of his duties, we need not have had to point out the fallacy of his facts, and the public would have been all the better served.

Having already expressed so favorable an opinion of Mr. Chadwick's Report, we may be permitted to observe that its great value does not consist in its containing anything wonderfully new either in principle or detail. The great importance and interest of the report arise from the authenticity and number of the facts. Some of these we shall notice, confining ourselves as much as possible to matters of practical value. It is quite impossible to condense the most general view of the facts contained in the report, so multifarious are they; much less to consider some general principles of political economy which Mr. Chadwick propounds. Our silence therefore must not be construed into an assent to, or dissent from, those principles, or even into an unqualified acknowledgment of the truth of all the alleged facts derived from his correspondents.

The Report commences with a demonstration of the extent of the evils which were the subject of inquiry. This is attained by a tabular return made up from the registration of the causes of death in England and Wales during the year 1838.

"A conception may be formed of the aggregate effects of the several causes of mortality from the fact, that of the deaths caused during one year in England and Wales, by epidemic, endemic, and contagious diseases, including fever, typhus and scarlatina, amounting to 56,461, the great proportion of which are proved to be preventible, it may be said that *the effect is as if the whole county of Westmoreland now containing* 56,469 *souls, or the whole county of Huntingdonshire, or any other equivalent district, were entirely depopulated annually, and were only occupied again by the growth of a new and feeble population* living under fears of a similiar visitation. The annual slaughter in England and Wales from preventible causes of typhus which attack persons in the vigour of life appears to be double in amount of what was suffered by the allied armies in the battle of Waterloo. . . . . But the number of persons who die is to be taken also as the indication of the much greater number of persons who fall sick, and who, although they escape, are subjected to the suffering and loss occasioned by the disease." (p. 3.)

The mortality in those attacked being 1 in 10, the numbers attacked are estimated at 250,000. The killed and wounded of smallpox are thus calculated:

"It appears that the extremes of mortality at the Smallpox Hospital in London amongst those attacked have been 15 per cent. and 42 per cent. But if, according to other statements, the average mortality be taken at 1 in 5, or 20 per cent., the number of persons attacked in England and Wales during the year of the return must amount to upwards of 16,000 persons killed, and more than 80,000 subjected to the sufferings of disease." (p. 4.)

If we were inclined to be hypercritical we should find ample scope for practice in these, the first statements of the Report. It is plain the statement we have marked by italics is an exaggeration—though an unintentional one. The deaths from epidemic diseases fall most extensively amongst children. In the year 1839, the deaths in Manchester from scarlatina, measles, hooping-cough and smallpox, were 1484; but only 132 of these were persons above five years of age, and only 16 above ten. Of 323 deaths from typhus, 101 were under fifteen years of age: so that there could not possibly be anything like the desolation Mr. Chadwick would have us suppose.

The general condition of the residences of the labouring classes where disease is found to be most prevalent is then exhibited. Reports from 11 cities and towns and from 17 districts, are laid under contribution. The extracts given by Mr. Chadwick deserve careful perusal. The close connexion between fever and the decomposing organic matter in open drains, sewers, privies, cesspools, &c., is clearly shown. The public arrangements external to the residences by which the sanatory condition of the labouring population is affected, are next reviewed under the several heads of town drainage; cleansing of houses, streets and roads; supplies of water, and land drainage. The details extracted from the voluminous reports are often curious. Some queer crotchety people have been the correspondents of the commissioners. The piety, patriotism, and profundity, of the provost of Inverness are only equalled by the niceness and naughtiness of his people. That gentleman writes:

"Inverness is a *nice* town, situated in a most beautiful country, and with every facility for cleanliness and comfort. The people are, generally speaking, a *nice* people, but their sufferance of nastiness is past endurance. Contagious fever is seldom or ever absent; but for many years it has seldom been rife in its pestiferous influence. The people owe this more to the kindness of Almighty God than to any means taken or observed for its prevention. There are very few houses in the town which can boast of either water-closet or privy, and only two or three public privies in the better part of the place exist for the great bulk of the inhabitants." (p. 43.)

In short, the people of Inverness are not very "nice," but a very social people, and worship Cloacina together under the wide canopy of heaven; in the streets as well as in the public penetralia.

Mr. Chadwick is a good tactician and makes a particularly happy hit when he demonstrates the great money-value of the refuse of towns. About 300 acres of poor land near Edinburgh, formerly dear at 40s. or 50s. per acre, have been irrigated during the present century by the contents of a large uncovered common sewer. This irrigation has so increased the value of the land that it lets for from £20 to £30 per acre. In 1826, £57 per acre was the rent of some of the richest meadows. As the sewer is a nuisance, attempts have been made to divert it. The parties interested have successfully resisted all these attempts, and estimate the value of the irrigation at £150,000. At this rate Mr. Chadwick makes the annual value of all the refuse of Edinburgh to be from £15,000 to £20,000. The value of the metropolitan refuse is nearly double the cost of all the water supplied to the metropolis. Science bids fair to make us all a sort of self-supporting mechanism; and to return our excretions in the shape of food as fast as they are eliminated: a consummation that will utterly rout the Malthusians, and make the human race eternal.

The whole of the observations in this section as well as the documents connected with it, in the appendix are important and interesting. Mr. Chadwick is quite at home when engaged with sewerage and drainage.

The "circumstances chiefly in the internal economy and bad ventilation of places of work; workmen's lodging-houses, dwellings, and the domestic habits affecting the health of the lower classes," are next reviewed. The working tailors seem to have more particularly attracted Mr. Chadwick's compassionate attention, and not without reason. We have occasionally witnessed the journeyman tailors of the metropolis at their work, and have always marvelled that they existed so long as they do. We are sorry we have not room for Mr. Brownlow's graphic description of the "tailoring" abominations. Poor fellows are described as fainting away, and tallow candles melting away from the excessive heat. And then the varieties of stenches! "It is not doubted," Mr. Chadwick observes, "by medical witnesses that one third at least of the healthful duration of adult life amongst milliners, dress-makers, and tailors, will be found to have been destroyed by the ignorance of the want of ventilation."

Mr. Chadwick undertakes to break a lance with Patissier about the health of tailors, and Mr. journeyman-tailor Brownlow officiates as his Sancho Panza. Mr. Chadwick has neglected to separate the facts of Patissier from his *à priori* inferences. His facts refer to tailors as they are, not as they might be, to diseased tailors, not to healthy; nor ought a few isolated instances of young tailors making good sailors and soldiers, and even grenadiers and dragoons, to be considered of much value. We would venture to assert that the young tailors so distinguished for their strength and courage were originally much more skilful boxers or poachers, than stitchers; and were oftener on their feet than their hams.

The effects of overcrowding of dwellings on the morals of the people, and of noxious physical agents on the personal condition and moral habits are well elucidated. We cordially agree with Mr. Chadwick in his opinions respecting the formation of drunken habits by the depressing influence (on the nervous system) of foul air and injurious employments. An irresistible craving for excitement is the leading effect of these influences. The Report of the Parliamentary Committee on drunkenness contains an exposition of Mr. Chadwick's views, repeated here.

Under the head "domestic mismanagement as a predisposing cause of disease," Mr. Chadwick touches upon the terrible subject of fever, its nature and origin.

"The medical controversy as to the causes of fever; as to whether it is caused by filth and vitiated atmosphere, or whether the state of the atmosphere is a predisposing cause to the reception of the fever, or the means of propagating that disease, which has really some other superior, independent, or specific cause, does not appear to be one that for practical purposes need be considered, except that its effect is prejudicial in diverting attention from the practical means of prevention." (p. 148.)

Without stopping to notice the want of precision in this sentence, or to ask Mr. Chadwick how he can prevent a disease without preventing its causes, and how he can prevent these without knowing them—we would simply suggest that the word *smallpox* be substituted for the word *fever* in the preceding passage; and read carefully over with this emendation. And the change is perfectly justifiable, so far, at least, as the Glasgow fever is implicated; that being, as is well known, an exanthem, resembling smallpox in the important circumstance that one attack affords immunity from a second. Mr. Chadwick apparently defines destitution to be a want of food—*starvation*; and then denies that destitution is a general cause of fever or facilitates its spread. We are of opinion that destitution is sometimes a cause of fever, always a most powerful auxiliary to its spread; but by destitution we mean the deprivation both of food *and* of all other requisites to a healthy condition of the body. To be destitute is to be crowded into miserable apartments, to want fresh air, fuel, clothes, water, soap, and all the common comforts of life. Mr. Chadwick evidently lays much stress upon the general fact stated by Dr. Davidson, that only 17 per cent. of the fever patients admitted into the Glasgow hospital from May 1st to November 1st, were spare and unhealthy. This fact proves nothing more than that the unhealthy and thin had already had the fever, or were acclimated, and were not liable to infection. The whole of Mr. Chadwick's arguments looks too much like special pleading in favour of hygienic measures, endangered by the fever controversy between Drs. Arnott and Alison. Mr. Chadwick steps in as arbiter, but *tantas componere lites* is not so easy a matter. Both the reporter and controversialists have been equally unmindful of an important fact we noticed in our review of Dr. Motard's essay, and to which we have again referred in our present Number in the article on Interments. It is this, that by constantly living in a poisonous vitiated atmosphere, people in towns may acquire an absolute or relative immunity from its ill effects; just as Europeans in the West Indies or America may become habituated to the causes of fever, or in other words, acclimated. This general fact utterly demolishes Dr. Alison's arguments, and enables us to state *à priori* that the effects of uncleanliness in towns are felt most severely by the unacclimated emigrants. Statistics confirm these inferences. Dr. Cowan found that of 178 inmates of the Glasgow Royal Infirmary, in April 1840, only 38 were natives of Glasgow, and 98 had not passed the prime of life there. Dr. Perry states that not more than 15 per cent. of patients admitted into the Albion-street Hospital were natives, and 25 per cent. had not been three years resident. The deaths from typhus will be found to exhibit a similar ratio.

*Table showing the comparative Viability of the Three great Classes of the Population in different Towns and Districts.*

| | Gentry and Professional Men. | | Tradesmen. | | Artisans, Operatives, Labourers, &c. | |
|---|---|---|---|---|---|---|
| | No. of Deaths. | Average Age of Deceased. | No. of Deaths. | Average Age of Deceased. | No. of Deaths. | Average Age of Deceased. |
| Rutlandshire . | — | 52 | — | 41 | —— | 38 |
| Wilts . . | 119 | 50 | 218 | 48 | 2061 | 33 |
| Derby . . | 10 | 49 | 125 | 38 | 752 | 21 |
| Bethnal Green . | 101 | 45 | 273 | 26 | 1258 | 16 |
| Kendal . . | 52 | 45 | 138 | 39 | 413 | 34 |
| Whitechapel . | 37 | 45 | 387 | 27 | 1762 | 22 |
| Kensington . . | 331 | 44 | 348 | 29 | 1258 | 26 |
| Leeds . . | 79 | 44 | 824 | 27 | 3395 | 19 |
| Strand . . . | 86 | 43 | 221 | 33 | 674 | 24 |
| Truro . . | 33 | 40 | 138 | 33 | 447 | 28 |
| Manchester . . | 137 | 35 | 1738 | 22 | 5597 | 15 |
| Liverpool . . | — | 38 | — | 20 | —— | 17 |
| Bolton . . | 103 | 34 | 381 | 23 | 2232 | 18 |
| Total. . | 1088 | 43 nearly | 4791 | 27⅗ | 19849 | 20½ |

We turn with pleasure from controversial matters to the interesting facts detailed by Mr. Chadwick in his inquiry into the "comparative changes of life in different classes of the community." The foregoing table is constructed from data in the Report. The average age of deceased is calculated on the deaths in the families generally of each class.

Thus the labouring population in Rutlandshire live (on the average) two and a half times longer than the labouring cellar-house population of Liverpool; but the gentry of Rutlandshire live three and a half times longer. The abbreviation of life experienced by all the labouring classes, but most heavily by those of it resident in towns, is not an abbreviation necessarily dependent on their employments. It is not, however, among the labouring classes only where life is thus unnecessarily cut short; the middle class suffers with them; and even the higher, but in a less degree, as is shown by the tables. It is no exaggeration to assert that a good system of medical police would raise the average life of the labouring class to the average of the trading; of the trading to that of the professional class; and of the professional class in towns to the average of the same class in rural districts; in short, that each might have life extended in duration one third at least beyond the present period. The influence of removable nuisances on the health of towns is admirably shown by two coloured maps. One is a map of Leeds by Mr. Baker, a surgeon in that town, and a gentleman who has brought much energy, industry, and intelligence to bear upon subjects connected with the public health; the other is a map of Bethnal Green. Mr. Baker distinguishes the undrained and uncleaned portions by a brown tint deepening in shade as the filth deepens. This map is not novel in its plan, but is not the less interesting on that account. It will be seen by reference to it, that the tracks of cholera and fever are nearly parallel.

A table on the map exhibits the favorable effects of good drainage, wide streets, and ample house-room, on the rate of mortality. The following table made up by Mr. Farr will be equally convincing to those acquainted with the metropolis.

"Mean annual mortality of females in twelve metropolitan districts during the two years and a half ending 31st December, 1839.

| *Districts.* | *Annual Deaths.* 1 in |
|---|---|
| Hackney | 57·87 |
| St. George, Hanover Square | 57·05 |
| Camberwell | 55·34 |
| Islington | 50·03 |
| Rotherhithe | 38·58 |
| Clerkenwell | 38·54 |
| St. Luke | 38·49 |
| Greenwich | 38·42 |
| St. George, Southwark | 33·77 |
| East and West London | 33·50 |
| St. Giles and St. George | 33·46 |
| Whitechapel | 28·15 |

(Report, p. 165.)

In our last Number we took occasion to place the principles of Malthus and Alison in antagonism, when noticing the "Principles of Population" of the latter. We stated that vice and misery were not checks on population; that they were, in fact, encouragers of population.

The poor-law commission was instituted on Malthusian principles; it was the last great act to which that writer's ideas gave birth. Yet we have here the fable of Saturn reversed, and the poor-law commissioners, with Mr. Chadwick as their organ, advocating anti-malthusian doctrines. It will be seen from the following tables that vice, shortness of life and misery and a rapid increase of the population are co-existent, and *vice versa*. The township of Broughton, we would premise, is inhabited almost exclusively by the families of the higher class connected with Manchester. The township of Cheetham and Crumpsall is also inhabited for the most part by the upper classes who live in peculiarly good houses with a good natural drainage.

| Localities. | Population. | | Deaths. | | Total Deaths of Males and Females. | Proportion of Births to Population. | Proportion of Illegitimate Births to Total Births. |
|---|---|---|---|---|---|---|---|
| | Males | Females | Males | Females | | | |
| | | | 1 in | 1 in | 1 in | 1 in | 1 in |
| Broughton . . . | 1554 | 2239 | 44·40 | 89·56 | 63·21 | 36·82 | 51·50 |
| Cheetham & Crumpsall | 3963 | 4862 | 45·03 | 63·14 | 53·48 | 34·74 | 50·80 |
| Pendleton . . . | 5109 | 5796 | 40·22 | 49·96 | 44·87 | 25·47 | 12·58 |
| Chorlton-upon-Medlock | 12551 | 15771 | 30·91 | 47·79 | 38·48 | 26·05 | 32·93 |
| Hulme . . . | 12850 | 13969 | 37·24 | 38·48 | 37·87 | 23·17 | 24·10 |
| Ardwick . . | 4586 | 5320 | 35·55 | 34·54 | 35·00 | 24·27 | 34·00 |
| Salford . . . | 24762 | 26760 | 27·30 | 36·60 | 31·42 | 22·83 | 21·90 |
| Manchester . . | 79061 | 84606 | 26·61 | 30·15 | 28·33 | 26·79 | 19·20 |
| Total . . . | 144436 | 159323 | 28·84 | 34·62 | 31·60 | 25·74 | 21·26 |

To make the proof of Mr. Alison's doctrines complete; and to exhibit the correctness of a principle we have before insisted on, namely, that a low physical condition is necessarily connected with a low moral condition, we subjoin the followingTable exhibiting the comparative mortality of ten registration districts in Leeds; with the ratio of births and illegitimate births to the deaths.

| Registration Districts. | Population. | Ratio of Deaths to the whole Population. | Ratio of Births to the whole Population. | Ratio of Illegitimate Births to Total Births. |
|---|---|---|---|---|
| | | 1 in | 1 in | 1 in |
| Chapeltown . . | 4538 | 57·7 | 30·6 | 74·0 |
| Whitkirk . . . | 3194 | 56·0 | 29·0 | 36·7 |
| Kirkstall . . . | 17816 | 45·6 | 24·8 | 23·1 |
| Rothwell . . . | 5557 | 45·1 | 28·2 | 24·6 |
| Wortley . . . | 16185 | 44·4 | 24·9 | 26·0 |
| Holbeck . . . | 16668 | 41·9 | 25·4 | 24·3 |
| Leeds, West . . | 32286 | 40·4 | 28·4 | 19·2 |
| Hunslet . . . | 15784 | 35·5 | 24·2 | 21·7 |
| Leeds, North . . | 30465 | 30·9 | 23·9 | 14·3 |
| East District (Kirkgate) . | 24862 | 28·8 | 24·3 | 20·0 |
| Total of Leeds . | 167355 | 37·3 | 25·5 | 20·1 |

It is such undeniable facts as these which display the great and solemn duties incumbent on the medical profession. To give increase of years to the labouring classes is to diminish crime, and empty prisons; to improve the morals and increase the comforts of the people; leading them at the same time to a practical enjoyment of the religion so eminently calculated to elevate the mind, and extend the enjoyments of the poor; in short, to work a greater and more beneficial revolution in England, than ever it has passed through.

Mr. Alison argues that a high degree of civilization and social happiness is the best check in population. This is proved in some degree by the experience of Geneva, deduced from the registries, commenced so early as the year 1549. We cannot afford space for the interesting tables quoted by Mr. Chadwick: but we may state that the probabilities of life have gradually increased in Geneva from 8½ years at the close of the sixteenth century, to more than five-fold, as follows: 8½, 13¼, 27¾, 31¼, 40⅔, 45, the latter being the present probability of life for each person born. In 1836, with a large proportion of adults the births barely replaced the deaths. Geneva having now attained a high degree of civilization and prosperity, the population is stationary.

Mr. Chadwick restates the statements made by writers respecting the physical deterioration of urban and manufacturing populations; and which we referred to particularly in our last Number. The race is deteriorated as the fecundity increases; but as there is a constant influx of healthy emigrants, the noxious agencies operating upon such populations do not display their full effect. We should infer, *à priori*, that if a cordon were drawn round the unhealthy districts of Manchester, so that this immigration were prevented, the inhabitants would, in time, be extinct; would become less in stature and numbers, and die away. The great extent to which this immigration takes place may be inferred from the fact stated by Mr. Chadwick, that in a late enumeration of the settled inhabitants of the labouring class in the lower parts of Westminster, it appeared that not more than one third of them were natives of London. The proportion is less in Glasgow.

Great fecundity in a population is not necessarily connected with great national strength. That is best shown, as Mr. Chadwick very correctly remarks, by the increased proportion of the adults who are of the age and strength for productive industry.

"M. Mallet bears testimony that the experience of Geneva is confirmatory of the important rule, that the strength of a people does not depend on the absolute number of its population, but on the relative number of those who are of the age and strength for labour. It is proved that the real and productive value of the population has there increased in a much greater proportion than the increase in the absolute number of the population. The absolute number of the population has only doubled in the instance of Geneva, during three centuries; but the value of the population has more than doubled upon the purely numerical increase of the population. In other words, a population of 27,000, in which the probability of life is forty years for each individual, is more than twice as strong for the purposes of production as a population of 27,000, in which the probability or value of life is only twenty years for each individual." (p. 185.)

There are several tables exhibiting the weakness of our population in some districts when contemplated from this point of view as compared with the population of other districts. We must refer the reader to the Report itself for these. Mr. Chadwick states a circumstance, noticed repeatedly during the late riots, that whenever a crowd is collected in a physically-depressed district, the small proportion of aged or even of the middle-aged and the great preponderance of youth is very striking. The following passage offers much matter for deep thought to the legislator and political economist.

"On expostulating on other occasions with middle-aged and experienced workmen on the folly as well as the injustice of their trade-unions, by which the public peace was compromised by the violences of strike after strike, without regard to the experiences of the suffering from the continued failures of their exertions for objects, the attainment of which would have been most injurious to themselves, the workmen of the class remonstrated with, invariably disclaimed connexion with the proceedings, and showed that they abstained from attendance at the meetings. The common expression was, they would not attend to be borne down by 'mere boys,' who were furious and knew not what they were about. The predominance of a young and violent majority was general." (p. 201.)

In this paragraph Mr. Chadwick expresses a great principle. A generation is swept away before it attains by suffering to a mature personal knowledge of what is to its social or political advantage; and is succeeded by another generation equally ignorant and quite as inexperienced. So rapid is the movement that neither history nor tradition can put in a warning. A juvenile population must of course be ruled like school-boys; and a numerous police is now, in fact, their ushers. Turn-outs are "barring-outs;" and a riot is "the school in an uproar." It would be madness to give universal suffrage to such a population. Hygienic measures, by increasing the average duration of life in manufacturing districts, would increase the numbers of efficient adult labourers, and so diminish the demand for an immigrant population. There would thus be a reflux on the men-growing agricultural districts, and improved methods of cultivation or emigration must provide for the surplus. It cannot be doubted that agricultural labourers and mechanics are much better colonists than stunted hand-loom weavers and pale-faced cotton-spinners; and it is equally certain that more populous colonies would extend and strengthen our empire, multiply our shipping and sailors, enlarge our commerce and manufactures; and facilitate emigration in proportion as more extended emigration becomes necessary. There is congestion now in the manufacturing portions of the body politic—congestion amounting almost to inflammation; medical police operating in the way indicated, would restore the balance of the circulation, and bring back a healthy state of the system.

Under the head of "pecuniary burdens created by the neglect of sanatory measures," Mr. Chadwick gives some convincing proofs of the costliness of our present let-alone system. The support of widows and orphans alone is no small charge in the poor-rates. During the year ending Lady-day, 1840, no fewer than 112,000 orphans, and 43,000 widows received relief from this national fund. Mr. Chadwick enters into an elaborate inquiry, and infers from various statistical facts, that nearly 27,000 cases of premature widowhood, and more than 100,000 cases of orphanage may be ascribed to removeable causes. A variety of interesting details are given under this head of pecuniary loss, to which we cannot even allude. We give the Dundee fever-bill for seven years, as an example. It is drawn up by the Rev. G. Lewis from statistical data. In the seven years mentioned, there were 11,808 cases of fever in Dundee, and 1312 deaths.

"Fever bill of Dundee from 1833 to 1839.

| | £ | s. | d. |
|---|---|---|---|
| Loss of labour for six weeks of 5248 adults, at 8s. per week | 12,595 | 0 | 0 |
| Attendance, medicine at home or Infirmary, at £1 each . | 5,248 | 0 | 0 |
| Loss of labour for six weeks of 5248 under age, at 4s. per week | 6,297 | 12 | 0 |
| Expense of treatment of the above at Infirmary or at home, at 10s. a piece . . . . . . | 2,624 | 0 | 0 |
| Loss by death of 656 adults at £150 each . . . | 98,400 | 0 | 0 |
| Loss by 656 deaths under age at £75 a piece . . . | 49,200 | 0 | 0 |
| Treatment of 1312 cases at £1 each . . . . | 1,312 | 0 | 0 |
| "Total expense for seven years | £175,676 | 12 | 0 |

"Or £25,026 13s. per annum." (p. 209.)

We next come to a vast variety of evidence of the effects of preventive measures in raising the standard of health and the chances of life. The costs of sickness and of prevention are compared, and some minute calculations are entered into which we fear some may consider as proving too much, and dismiss with one or other of those contemptuous monosyllables in which pithy people delight. The inability of workmen to improve their own condition, and the necessity of extrinsic aid for obtaining this improvement are satisfactorily exhibited. Here again Mr. Chadwick shows himself a sound-thinking anti-Malthusian. The employers' influence on the health of their workpeople is well shown, and proved to be exceedingly great. A very happy example of the modes in which it can be easily and successfully exercised is furnished by the mills at Catrine, in Ayrshire, under the superintendence of Mr. Archibald Buchanan. Mr. Chadwick discusses at length the materials and methods used in building cottages, and indicates those which are to be avoided and preferred. Several plans and drawings illustrate this portion of the text. The discussion respecting common lodging-houses (section viii.) might have been brought in here. An inquiry into the effects of public walks and gardens on the health and morals of the lower classes ends the section, and a plan of the *arboretum* at Derby, the patriotic gift of Mr. Strutt to his native town, is placed in the Appendix. A walk of two miles is contrived in eleven acres, and 1000 specimens of shrubs and plants are displayed.

The seventh section of the Report is occupied with a discussion of the recognized principles of legislation, and the state of the existing law for the protection of the public health. We are inclined to look on this as the best-considered section in the whole Report. Mr. Chadwick is quite at home amongst drains and sewers, as we have remarked before. A very good case is made out for legislative interference; and we anticipate the staunch support of the most intelligent and influential owners of property in favour of some such legislative measures as he proposes. We quote the following passage; firstly, because it contains truth, and, secondly, (and this is Mr. Chadwick's opinion,) because the truth is equally applicable to a system of medical police, as to a system of drainage and road-management:

"A competent, scientific, and efficient management, let it be applied to what part of these works it may, can scarcely fail to be immediately as well as ultimately the most economical management. ....... .... Division of labour in the arts derives its efficiency from combination, adaptation, and subordination to direction to one end." (p. 323.)

The concluding division of this section is an essay on boards of health and medical police. It will be seen that Mr. Chadwick puts his literary charger to anything. Engineering and doctoring might be called stiff fences for a barrister; but over them both our stout-hearted reporter vaults with perfect nonchalance. It is a singular psychical phenomenon, that a member of the legal profession, after touching, of course, only the surface of the science of medicine, should be able to come forth as if his life had been devoted to the subject. Mr. Chadwick, after grinding in the medical mill with Messrs. Kay, Arnott, Southwood Smith, and others for a while, appears here in a dress as professional as that of his instructors. The inefficiency of boards of health and their failure in Ireland is stated. The importance of the functions of medical officers in connexion with executive authority; the means and economy of skilled services for the prevention of disease; the administrative means for promoting the extension of medical science, and the necessity of attention to the prevention of disease are some of the questions discussed. But Mr. Chadwick shall speak for himself on these subjects:

"The action of a board of health upon such evils as those in question must depend upon the arrangements for bringing under its notice the evils to be remedied. A body of gentlemen sitting in a room will soon find themselves with few means of action, if there be no agency to bring the subject matters before them; and an inquiring agency to seek out the evils from house to house, wherever these evils may be found, to follow on the footsteps of the private practitioners, would be apparently attended with much practical difficulty." (p. 341.)

After giving evidence as to the loathsome condition of the lowest class of residences, Mr. Chadwick observes:

"It has only been under the strong pressure of professional duties by the physicians and paid medical and relieving officers, responsible for visiting the abodes of the persons reduced to destitution by disease, that the condition of those abodes in the metropolis have of late been known; and I believe that it is only under continued pressure and strong responsibilities and interests in prevention, that investigation will be carried into such places, and the extensive physical causes of disease be effectually eradicated. Whilst experience gives little promise even of inquiries from such a body as boards of health without responsibilities, still less of any important results from the mere representations of such bodies separated from executive authority, I would submit for consideration what appears to me a more advantageous application of medical science, viz., by uniting it with boards having executive authority." (p. 343.)

Here, then, Mr. Chadwick fairly pledges himself to a plan of medical police. The basis of this plan is the Poor Law Commission. The 2300 union surgeons are to make the necessary hygiënic inquiries in connexion with the relieving officers. These individuals are to be, in fact, perpetually perambulating "annoyance juries," while the boards of guardians will be constituted hebdomadal courts leet. Some practical evidence of the probable working of the plan is given:

"From the consideration of such practical evidence, it will be seen that the ordinary duties of the relieving officer in the first instance, and of the medical officer afterwards, ensure domiciliary inspection of large districts to an extent and with a degree of certainty that could scarcely be ensured or expected of any agents or members of a board of health unconnected with positive administrative duties. The inspection of these officers of the boards of guardians more than supplies the external inspection of inquests or of the leets; and it is submitted that in their position these boards may most beneficially exercise the functions of the leet in reclaiming the execution of the law, as against acts of omission and of commission, by which the poorest of the labouring classes are injured and the ratepayers burdened. It may therefore be submitted, as an eligible preliminary general arrangement, that it shall be required of the medical officer as an extra duty, for the due performance of which he should be fairly remunerated, that on visiting any person at that person's dwelling, on an order for medical relief, he shall, after having given such needful immediate relief as the case may require, examine, or cause to be examined, any such physical and removable causes as may have produced disease or acted as a predisposing cause to it; that he shall make out a particular statement of them, wherein he will specify any things that may be and are urgently required to be immediately removed. This statement should be given to the relieving officer, who should thereupon take measures for the removal of the nuisance at the expense of the owner of the tenement, unless he, upon notice which shall be given to him, forthwith proceed to direct their removal." (p. 348.)

Mr. Chadwick would not permit the delay of a day, if the medical officer should think delay dangerous, except in cases of appeal, or where a higher expense than £5, or a year's rent was involved.

"It is not unfrequent," Mr. Chadwick asserts, "to hear the expression of a wish from these officers, that some person unconnected with the district may be sent to examine the afflicted place and initiate the proper proceedings." This assertion "initiates" us into part the second of Mr. Chadwick's plan, namely, the appointment of *District Medical Officers.* These are to be for the special aid and supervision of the established medical relief, and for other duties. But Mr. Chadwick shall speak for himself:

"It will frequently be found that there is the like need of immediate local inspection of the medical treatment of the destitute, that there is of a grade of inspecting surgeons for the military hospitals. It cannot be otherwise than that amidst a numerous body of men there must be much error and neglect in the treatment of the destitute, in the absence of immediate securities against neglect. The most able of the guardians would confess that if they are not entirely incompetent to supervise medical service, they are at the best but imperfectly qualified for such a task, and the medical officers would act with more satisfaction to themselves from the supervision of officers from whom they might derive aid and confidence. But besides the medical treatment of the inmates of the workhouses and prisons, there are other cases within most districts which need the preventive service of a superior medical officer for the protection of the public health." (p. 350.)

We are not quite sure that we perfectly comprehend all that the last sentence is meant to imply. We suppose, however, that the district medical officers will inspect prisons (mentioned for the first time) as well as workhouses, and supervise the medical treatment of prisoners as well as of paupers. The "other cases" are

"First in the cases where the poorer classes are assembled in such numbers as to make the assemblage *quasi* public, and afford facilities for medical inspection, as in schools. Secondly, also in places of work and in workmen's lodging-houses. The occasional visits of a district officer for the prevention of disease would lead to the maintenance of due ventilation, and to the protection of the workpeople on such points as are already specified as injurious to the health, and that arise simply from ignorance, and are not essential to the processes. An examination of such places, if only quarterly, would lead to the most beneficial results." (p. 350.)

Mr. Chadwick would intrust the duties of sub-inspector of factories to these district officers, and thus he thinks a sanatory supervision of the labouring classes would be secured:

"There would still remain, however, those of the labouring classes who do not work or lodge in large numbers, or work in a *quasi* public manner, to bring them within the means of convenient inspection. There would also remain without protection the cases of persons of the middle classes. To meet these cases I would suggest that the information brought to the superintendent-registrar as to the cause of death, imperfect and hearsay as it yet is, may serve as the most accurate index to the direction of the labours of a district officer appointed to *investigate the means of protecting the health of all classes*. .......... For example, wherever, on the examination of these registries, deaths from fever or other epidemics were found to recur regularly, and in numbers closely clustered together, there will be found, on examination, to be some common and generally removable cause in active operation within the locality." (p. 352.)

The mortality of trades and the causes of that mortality could be investigated by the same means. We think this is a very happy hit of Mr. Chadwick's. He justly adds:

"One of the most important services, therefore, of a superior medical officer of a district, would be to ensure the entries of the causes of death with the care proportioned to the important uses to be derived from them. The public should be taught to regard correct registration as being frequently of as much importance for the protection of the survivors as a post-mortem examination is often found to be." (p. 353.)

Mr. Chadwick next proceeds to argue, that a combination of medical services in one scientific individual, would secure both greater economy and efficiency. Of this there can be no doubt. Mr. Chadwick refers particularly to the medical service and inspection of prisons; the inspection of lunatic asylums and of recruits; to union surgeons; to the granting of medical certificates; to factory children; and to medical evidence at coroner's inquests. These and other services, it is observed, are divided in such portions as only to afford remuneration in such sums as £40, £50, £60, or £80 each; and many smaller and few larger amounts. Mr. Chadwick observes that a fee of a guinea is given at each inquest for medical evidence. This is certainly a mistake. There are many inquests held without any medical evidence being demanded. Probably if the fees paid to medical witnesses at assize trials be included with the fees paid at inquests, the medical evidence in criminal cases may cost the country £12,000 or £15,000 per annum. The medical attendance at the prisons, under the authority of the inspectors of prisons, cost, in the year 1838, the sum of £8890 15s. We have made out the amount from the returns for that year. The following is sound doctrine and well expressed; and we think Mr. Chadwick feels the truth of what he writes; let but the government act on such principles and the support of the profession is secured.

"Whatever may be yet required for placing the union medical officers on a completely satisfactory footing, the combination of the services of several parish doctors in the service of fewer union medical officers will be found to be advances in a beneficial direction. The multiplication or the maintenance of such fragmentitious professional services is injurious to the public and the profession. It is injurious to the profession by multiplying poor, ill-paid, and ill-conditioned professional men. Although each may be highly paid in comparison with the service rendered, the portions of service do not suffice for the maintenance of an officer without the aid of private practice; they only suffice, therefore, to sustain needy competitors for practice in narrow fields. Out of such competition the public derive no improvements in medical science, for science comes out of wide opportunities of knowledge and study which are inconsistent with the study to make interests, and the hunt for business in poor neighbourhoods...........The highest medical authorities would agree that, whatsoever administrative arrangements sustain narrow districts and narrow practice, sustain, at a great public expense, barriers against the extension of knowledge by which the public would benefit, and that any arrangements by which such districts or confined practice is newly created, will aggravate existing evils. An examination of the state of medical practice divided amongst poor practitioners in the thinly-populated districts, shows that, but for the examinations, imperfect though they be, as arrangements which sustain skill and respectability, a large part of the population would be in the hands of ignorant bonesetters." (p. 355.)

We will now briefly recapitulate what is comprised in Mr. Chadwick's plan of medical police. As we understand it, it is intended to secure, 1, medical attendance on the sick poor and sick prisoners; 2, medical inspection of streets and houses and of the sewers, drains, privies, &c. appertaining to them; 3, medical supervision of union surgeons: 4, medical inspection of recruits and factory children; 5, medical inspection of prisons, workhouses, lunatic asylums, schools, manufactories, workshops, and other *quasi* public establishments; 6, the investigation of the causes of disease and the supervision of the mortuary registers; 7, the practice of forensic medicine so far as it involves the supplying of medical evidence at coroners' inquests. Mr. Chadwick may possibly not purpose to accomplish all this; we are certain, however, that we have omitted no part.

Those of our readers who may have traced with us the extent of public hygiène as developed in one of our recent Numbers, will see that, as a beginning and fragment of a system, Mr. Chadwick's plan is exceedingly meritorious; as the outline of a complete system, it is exceedingly defective. As, however, we hope on some future occasion, to discuss all the bearings of this most important subject at full length, we will here only guard ourselves against being supposed to subscribe to all Mr. Chadwick's plans as the best that could be contrived, while expressing our conviction that, if adopted exactly as proposed by him, they would be a boon of incalculable value to the public.

We think the consolidation of public medical offices is one of the best parts of the plan. The opinions of Du Châtelet (quoted approvingly by Mr. Chadwick) respecting the qualifications of public medical officers are well-founded.

"It is generally thought in the world that the medical knowledge acquired in the schools is all that is necessary to become a useful member of the council. The greater part of medical men themselves share this opinion: and on the strength of some precepts which they have collected from books on health and professions, they think themselves sufficiently instructed to decide on the instant the gravest questions, which can only be resolved by special studies. A man may have exhausted medical literature; he may be an excellent practitioner at the sick-bed, a learned physician, a clever and eloquent professor; but all these acquirements, taken in themselves, are nearly useless in a *conseil de salubrité* like that of Paris. .......... It requires habit and the frequenting of the places of work." (App. p. 423.)

M. Du Châtelet insists, in fact, that a theoretical and practical knowledge of the subject is necessary; and Mr. Chadwick adopts the same views. This principle should be kept steadily in view in establishing a system of public hygiène. Care should be taken, however, that a practical knowledge of disease be combined with this practical knowledge of hygiene. It is obvious that, if an officer of public health be debarred from private practice, he is shut out from the best—indeed the only—field for the acquisition of the necessary practical knowledge both of the diseases of the poor, and of the causes of disease removable by hygiènic measures. This practical knowledge of details would also be the best guarantee for the judicious performance of the duties of the higher grade, the district medical officer. No officer, therefore, should attain the higher grade except by service in the lower.

But our limits are reached, and we must here conclude without noticing at all the local reports; to these we hope to return at a future period. We cannot, however, close this article without once more expressing our approval of the talent, public-spirit, diligence, and enlarged views of Mr. Chadwick. Nothing but the enthusiasm of a high philanthropy could have prompted him to undertake the laborious task which he has so admirably executed. He will find his appropriate reward in the consciousness of being the author of great good to his fellow-men.

---

